# Organ-Sparing Surgery for Testicular Germ Cell Tumors: A Current Perspective

**DOI:** 10.3390/medicina59071249

**Published:** 2023-07-05

**Authors:** Esther García Rojo, Gianluca Giannarini, Borja García Gómez, Javier Amalio Feltes Ochoa, Félix Guerrero Ramos, Manuel Alonso Isa, Ricardo Brime Menendez, David Manuel Saenz Calzada, Juan Justo Quintas, Agustín Fraile, Celeste Manfredi, Javier Romero Otero

**Affiliations:** 1 Department of Urology, Hospital Universitario HM Sanchinarro, 28050 Madrid, Spain; esthergrojo@hotmail.com (E.G.R.); felix.guerrero@rocclinic.com (F.G.R.); ricardo.brime@rocclinic.com (R.B.M.); jjustoquintas@rocclinic.com (J.J.Q.); 2 ROC Clinic Direction, Martínez Campos 17, 28010 Madrid, Spain; bgarciagomez@rocclinic.com (B.G.G.); javier.feltes@rocclinic.com (J.A.F.O.); manuel.alonso@rocclinic.com (M.A.I.); david.saenz@rocclinic.com (D.M.S.C.); agustin.fraile@rocurologia.com (A.F.); 3 Urology Unit, Santa Maria della Misericordia University Hospital, 33100 Udine, Italy; g.giannarini@gmail.com; 4 Department of Urology, Hospital Universitario HM Montepríncipe, 28660 Madrid, Spain; 5 Department of Urology, Hospital Universitario HM Puerta del Sur, 28938 Madrid, Spain; 6 Department of Urology, Hospital Universitario HM Rivas, 28521 Madrid, Spain; 7 Unit of Urology, Department of Woman, Child and General and Specialized Surgery, University of Campania “Luigi Vanvitelli”, 81100 Naples, Italy; manfredi.celeste@gmail.com

**Keywords:** testicular cancer, testicular tumors, organ sparing, surgery

## Abstract

*Background and Objectives:* We aimed to evaluate the oncological and functional outcomes of organ-sparing surgery for testicular germ cell tumors, a procedure that seeks to strike a balance between effective cancer control and organ preservation, in the treatment of testicular tumors. We aimed to discuss the surgical technique and complications, and determine the appropriate candidate selection for this approach. *Material and Methods:* A comprehensive literature search was conducted to identify relevant studies on organ-sparing surgery for testicular tumors. Various databases, including PubMed, Embase, and Cochrane Library, were used. Studies reporting on surgical techniques, complications, and oncologic and functional outcomes were included for analysis. *Results:* Current evidence suggests that organ-sparing surgery for testicular germ cell tumors can be considered a safe and efficacious alternative to radical orchiectomy. The procedure is associated with adequate oncological control, as indicated by low recurrence rates and low complication rates. Endocrine testicular function can be preserved in around 80–90% of patients and paternity can be achieved in approximately half of the patients. Candidate selection for this surgery is typically based on the following criteria: pre-surgery normal levels of testosterone and luteinizing hormone, synchronous or metachronous bilateral tumors, tumor in a solitary testis, and tumor size less than 50% of the testis. *Conclusions:* Organ-sparing surgery for testicular germ cell tumors offers a promising approach that balances oncological control and preservation of testicular function. Further research, including large-scale prospective studies and long-term follow-ups, is warranted to validate the effectiveness and durability of organ-sparing surgery and to identify optimal patient selection criteria.

## 1. Introduction

Testicular cancer is a rare form of cancer that accounts for less than 1% of all male cancers [1]. However, it is the most common cancer among young men aged 15 to 35 years old [2]. The incidence of testicular cancer has been increasing worldwide, with the highest rates observed in Northern Europe [3].

There have been numerous previous classifications of testicular tumors from a variety of panels and organizations. For many years, the British testicular tumor panel was widely used in many countries [4]. Currently, the most widely used classification is that of the World Health Organization (WHO), which was updated in 2022 [5].

The standard treatment for testicular cancer is radical orchiectomy, which involves removing the entire affected testicle [6]. This procedure is highly effective in curing the disease, but it also has significant drawbacks. Radical orchiectomy is associated with a high risk of psychological distress, sexual dysfunction, and infertility, which can have a significant impact on a young man’s quality of life [7,8,9,10].

Organ-sparing surgery, also known as testis-sparing surgery or partial orchiectomy, is a surgical approach that aims to remove the tumor while preserving the healthy tissue and the function of the testis. Organ-sparing surgery has gained increasing popularity in recent years as an alternative to radical orchiectomy for select cases of testicular tumors, with growing scientific evidence [9,11,12,13,14].

Testis-sparing surgery (TSS) is a guideline-recommended treatment option for patients with small non-palpable tumors, synchronous or metachronous bilateral testicular germ cell tumors (GCTs), or GCT in a solitary testicle [15]. The benefits of this surgery seem numerous: by preserving the healthy testicular tissue, organ-sparing surgery minimizes the risk of infertility, sexual dysfunction, and psychological distress associated with radical orchiectomy [16]. It also allows young men to maintain their endocrine function and testosterone production, which can have important implications for their overall health and well-being [14,15]

This manuscript reviews the latest scientific evidence on testicular-sparing surgery for the treatment of testicular cancer, its surgical modalities, and its oncological and functional impact.

## 2. Materials and Methods

A comprehensive literature search was performed, using MEDLINE, EMBASE, and Cochrane Central Controlled Register of Trials (CENTRAL) databases. Medical Subject Heading (MeSH) terms and keywords such as “testicular cancer”, “organ-sparing surgery”, “partial orchiectomy”, “testis-sparing surgery”, “testicular tumor”, “oncological outcomes”, and “functional outcomes” were used. No date limits were imposed. Case series as well as review papers were included. The search was restricted to English papers. Animal studies were excluded.

The reviewers team favored the inclusion of articles from the last 10 years to give up-to-date information, although they did not exclude older highly referenced reports. The reference lists of articles identified by this search strategy were also reviewed, and the working group selected relevant references. The authors reviewed the relevant published literature up to March 2023 for inclusion in this narrative review. Each publication was reviewed by at least two reviewers and when there was no consensus a third reviewer decided on its inclusion.

## 3. Results

### 3.1. Patient Eligibility

The use of TSS for testicular malignancies remains a matter of debate, particularly in cases where the other testis is normal. However, the urological community is showing an increasing interest in organ-preserving surgery as a viable substitute for radical orchiectomy in many instances.

As per the German Cancer Study Group’s indications, TSS could be a potential choice for select patients with malignant tumors in a solitary testis or bilateral tumors, provided that the lesion’s diameter is less than 2 cm, there is no invasion of the rete testis, and the preoperative serum luteinizing hormone (LH) levels are normal. In such scenarios, the removal of the mass should be accompanied by multiple biopsies of the surrounding tissue and adjuvant radiotherapy should be considered for seminomas [17].

On the other hand, TSS has been broadly utilized in the treatment of benign tumors, with testicular ultrasound (US) and intraoperative frozen-section examination enabling the surgeon to confirm the benign nature of the testicular mass with near certainty, effectively reducing the risk of recurrence to virtually zero [18,19,20,21].

The size of the mass appears to be a critical factor in considering elective TSS; various studies have suggested that for nonpalpable, asymptomatic masses with diameters of less than 2 cm, TSS could be the most suitable approach, given that the probability of benign histology is about 80% [22,23,24,25,26,27]. Until recently, the threshold for tumor volume was 30% of total testicular volume, but this could be increased to a maximum of 50% if serum testosterone and LH concentrations are within normal limits to exclude compensated Leydig cell failure [25,28].

Furthermore, TSS is increasingly accepted in pediatric surgery due to the higher incidence of benign tumors. This method is particularly beneficial in younger children, as it can minimize the risk of late-onset hypogonadism and provides significant physiological and cosmetic advantages [29,30,31].

According to the guidelines of the European Association of Urology (EAU), testicular-sparing surgery is considered a viable option for patients with suspected benign tumors or uncertain masses with negative tumor markers. However, they emphasize that radical orchidectomy is the preferred standard of care for patients with likely malignant testicular tumors. Nonetheless, in cases of synchronous bilateral tumors or tumors in a single testis, testicular sparing surgery can be considered, but only in conjunction with frozen section examination. These guidelines note that it is important for patients to understand that there is limited data on the oncological safety of testicular-sparing surgery, and local recurrence rates can be as high as 26.9%. Additionally, patients should be informed about the potential need for radiotherapy if the histology reveals evidence of germ cell neoplasia in situ (GCNIS). The guidelines also stress that testicular-sparing surgery should only be performed in experienced medical centers [32].

The American Urological Association (AUA) guidelines propose TSS as an alternative to radical orchiectomy in patients with tumors < 2 cm, having ambiguous US/physical exam results and negative tumor markers, a solitary testis, or bilateral synchronous tumors. However, patients must be advised on potential risks such as higher recurrence, the regular monitoring necessity, the role of adjuvant radiotherapy in reducing local recurrence, the effect of radiotherapy on sperm and testosterone production, and the possibility of testicular atrophy necessitating testosterone replacement therapy or potential subfertility/infertility. Biopsies of the normal testicle tissue are advised during TSS. A trans-scrotal approach is cautioned against due to higher recurrence rates [33].

Table 1 provides a summary of the criteria that patients must meet to be eligible for organ-sparing surgery for testicular tumors.

### 3.2. Surgical Technique

The first detailed presentation of this surgical technique was given by Stoll et al. in 1986 [34]. They described how high-frequency ultrasound could be used as a guidance system for the enucleation of a non-palpable Leydig cell tumor. The methodology evolved over time, culminating in 2002 when Hopps and Goldstein standardized the procedure [35]. They introduced a magnification system to enhance the detection and thorough removal of small, non-palpable lesions.

Organ-sparing surgery for testicular tumors is not without its challenges. The main challenge is ensuring that the surgery removes all cancerous tissue while preserving the healthy tissue. This requires skilled surgeons and careful planning before and during the procedure. Another challenge is ensuring that the remaining healthy testicular tissue functions normally and does not develop complications such as atrophy or fibrosis [19].

It is generally recommended to use a conventional inguinal approach when performing a TSS to prevent any disturbance to the scrotum [36]. Some authors suggest that clamping the cord before bringing the testicle into the operative area can reduce the risk of tumor spread due to manipulation, but there is scant literature to support this traditional practice. The use of clamping, and whether it should be done under cold or warm ischemia conditions, remains a topic of debate. In a large study by Leonhartsberger et al., 65 patients underwent radical orchiectomy and TSS without early cord clamping and the authors reported that no patients showed signs of disease after a median follow-up period of 52.5 months [13].

If TSS is to be performed, a crosswise cut in the tunica albuginea is typically made after the testicle has been exposed, which allows for identification of an avascular plane [15]. Upon exposure, the tumor is typically surrounded by a pseudocapsule, enabling it to be easily distinguished from the normal parenchyma; however, a challenging extraction might suggest malignant infiltration. If the tumor is not physically detectable, tools such as an ultrasound or a microscope can be used to locate the lesion, followed by the removal of an excision margin of 2–5 mm [26,36]. The tumor that has been removed, along with any additional collected samples from the tumor site, are then put through a frozen section examination. This examination is used for histological identification of the tumor, detection of GCNIS, and confirmation of whether the surgical margins are positive [37].

A microsurgical approach, involving an operating microscope, can be employed if the necessary equipment is accessible and the surgeon possesses the required skills. The primary reason for utilizing a microsurgical technique is typically to address a nonpalpable tumor that has been detected via ultrasound [22,25]. Another situation where this approach might be used is in cases where a man with azoospermia is undergoing testicular sperm extraction (TESE) for assisted reproductive methods and incidental testicular abnormalities are discovered on ultrasound [38]. Proponents of microsurgical techniques assert that it can offer several advantages, such as enhanced preservation of healthy tissue and reduced risk of damaging the tunica albuginea’s vasculature. These benefits could theoretically decrease the risk of hypogonadism, testicular atrophy, and infertility.

De Stefani et al. conducted the most extensive study of testis-sparing surgeries using a microsurgical approach, involving 23 patients. Tumors were nonpalpable and showed no evidence of elevated tumor markers. One patient underwent a second operation five years after the initial surgery, as the initial pathology reported normal testicular tissue. The subsequent procedure revealed seminoma, necessitating a radical orchiectomy. None of the patients in this study experienced disease progression. All were free of disease and exhibited normal scrotal ultrasound results after an average follow-up period of 35 ± 25 months [25].

However, because each of these surgical techniques lacks concrete evidence supporting their functional or oncological benefits, it is our belief that the choice of surgical approach should be based on the surgeon’s individual preference [9].

### 3.3. Complications

Organ-sparing surgery for testicular tumors is associated with a low risk of complications, with rates ranging between 1% and 6% across different studies [26,28]. The most common complications include bleeding/hematoma, infection, and testicular atrophy.

In the systematic review conducted by Favilla et al., which incorporated data from a total of 725 patients from 26 studies that reported this outcome, the authors reported a complication rate of 2%. Specifically, hematoma was observed in seven patients (1.2%), necessitating surgical intervention for hemostasis in one case. Additionally, testicular atrophy was noted in four patients (0.7%), and a single case (0.2%) of inflammation was reported during the postoperative period [39].

Heidenreich et al., in their systematic review published in 2023, concluded that severe complications following TSS are rare and are observed in less than 1% of patients [28].

### 3.4. Oncological Outcomes

The first successful case of TSS was performed by Richie in 1984, who employed this approach for a patient with synchronous bilateral seminoma. Remarkably, the patient remained disease-free without requiring permanent androgen replacement even after a 2.5-year follow-up. However, the author referred to this treatment strategy as “unorthodox” [40].

Since then, several series and case reports have described TSS for selected patients with testicular GCTs (organ-confined tumors in patients with synchronous bilateral tumors or solitary testis with normal preoperative endocrine function).

The most comprehensive series on TSS for malignant tumors was published by The German Testicular Cancer Study Group. The successful application of TSS was noted in 101 patients across eight high-volume institutions with either bilateral GCTs or a solitary testis GCT. The average tumor diameter was reported as 15 mm (ranging between 5–30 mm). GCNIS was discovered in 84% of the cases, with 79% of these patients receiving adjuvant radiation with 18 Gy. After a median follow-up of 80 months, 100 patients remained disease-free. Local recurrence was observed in six patients, all of whom were successfully treated with inguinal orchiectomy [17,41].

Bojanic et al. reported on 24 patients who underwent TSS for bilateral GCTs or solitary testis tumors. All tumors were less than 2 cm in diameter. Of these, seven patients experienced local recurrence but were successfully treated with either radical orchiectomy or a second TSS. The overall survival rate of the study group was 100% at a median follow-up of 51 months [42].

In another study by Steiner et al., TSS was performed on 11 patients with GCTs. All tumors were less than 25 mm in diameter, and 10 of them were diagnosed concurrently with ipsilateral GCNIS. One local recurrence was observed and TSS was repeated with subsequent local radiation. All patients were disease-free at an average follow-up of 46.3 months [43].

The management of GCNIS is of critical importance as the majority of untreated GCNIS cases will develop into invasive disease. The presence of GCNIS in a testis carries an estimated risk of evolving into invasive disease of 50% within 5 years and 70% within 7 years [44]. Therefore, it is necessary to consider local radiotherapy for patients with GCNIS, particularly for those with a solitary testis [45,46,47]. In Avuzzi et al.’s study, radiotherapy following testicular-sparing surgery showed no local or distant relapses in a medium-term follow-up, with hormonal function preserved in about 54.5% of patients. Associations were noted between baseline testosterone levels, tumor size, and risk of exogenous androgen replacement [46]. Dieckmann et al. highlight that 18–20 Gy local radiotherapy eradicates the majority of GCNIS. However, their study also emphasizes the potential for treatment failure, evidenced by cases of relapse occurring over a decade post-treatment. The failure rate is estimated to be around 1% [45].

There are different systematic reviews published over the years (Table 2), with different criteria for inclusion. In the one published by Ory et al. in 2021, in which no meta-analysis is carried out due to the heterogeneity of the studies (retrospective noncontrolled studies), they conclude that TSS is a safe and efficacious technique with regards to oncological control and postoperative hormonal function and should be given serious consideration in cases of nonpalpable, small tumors under 2 cm and in men with bilateral tumors or with solitary testicles [9].

In the systematic review conducted by Favilla et al. in 2021, which incorporated data from 26 studies and 603 patients, it was noted that the local recurrence rate was reported at 3.48%. Furthermore, the overall recurrence rate varied, with figures ranging from 0% to 26.9% for malignant cases, and from 0% to 0.1% for benign lesions. It is important to note that a meta-analysis was not performed due to the diversity of the included studies. The authors concluded that TSS is a safe and feasible method when applied in accordance with specific guidelines. They suggested that TSS should be considered as a significant tool for managing testicular tumors in selected and well-informed patients.

In the recent study published by Grogg et al. in 2022, which encompasses 32 studies and offers data from 285 patients, it was reported that 87% of the patients who underwent TSS experienced no relapse after a median follow-up period of 38 months. Meanwhile, local recurrence was documented in 13% of patients after a median duration of 12 months. Distant recurrence post-TSS was observed in 2% of the patients following a median period of 19 months. The authors conclude that TSS should only be offered to well-informed patients with a singular testicle, a singular tumor less than 2 cm located at the lower pole of the testicle, and normal preoperative endocrine function. Unless patients plan to father a child within a short time frame, adjuvant testicular radiotherapy should be recommended [11].

Finally, in the most recent systematic review published by Heidenreich et al. in 2023, data from eight studies, comprising a total of 252 patients, were evaluated. The authors reported a local recurrence rate ranging from 4–6% to 15.9%. The distant recurrence rate fell within the 2–4% range. In terms of cancer-specific survival, figures approached a near-100% rate, reinforcing the effectiveness of the treatment strategies evaluated. This review further emphasized the significance of adjuvant radiation therapy in preventing local relapses. In fact, the authors reported that oncological outcomes were excellent when this treatment strategy was incorporated. It was also noted that the local recurrence rate might increase to 4–6% should adjuvant radiation therapy be omitted. The results of this review contribute to the accumulating evidence that supports the use of TSS in combination with adjuvant radiation therapy, further emphasizing the importance of rigorous patient management strategies in the treatment of testicular tumors [28].

The protocol for follow-up after TSS remains unclear and hasn’t been extensively researched in existing literature. Therefore, careful patient selection and frequent follow-ups incorporating ultrasound are necessary until better protocols are established.

### 3.5. Functional Outcomes

One of the main objectives of TSS for GCT is to preserve endocrine function [28].

There is an elevated risk of early-onset hypogonadism in patients with testicular GCTs. This condition might be further exacerbated by supplementary local radiation therapy [7,8].

In the study by The German Testicular Cancer Study Group with a total of 101 patients, 84 (over 83%) maintained normal testosterone serum levels after an average follow-up of 84 months. Of the remaining patients, six already had low serum testosterone preoperatively and the rest exhibited new-onset hypogonadism requiring testosterone supplementation postoperatively [16]. Factors like tumor size exceeding 2 cm, increased serum LH, and TSS with warm ischemia were associated with postoperative hypogonadism. Therefore, meticulous patient selection and proficient surgical technique are crucial.

In the work published by Steiner et al., only one out of 12 patients displayed hypogonadism after an average follow-up of 60 months [43].

The meta-analysis of Patel et al. revealed a 9.7% risk of hypogonadism development. They inferred that significant tumor volume and poor preoperative hormonal status are the main risk factors for postoperative hypogonadism [16].

On the other hand, Grogg et al.’s review revealed a 27% incidence of hypogonadism. However, it lacked information on preoperative endocrine function and the size of the removed lesion. Thus, no conclusions about hypogonadism prevention could be derived [11].

Although it is well established that most men with GCT and GCNIS suffer from subfertility or infertility because of azoospermia or significantly diminished spermatogenesis, another goal of TSS is fertility preservation [49,50,51]. In cases of synchronous tumors or a tumor in a single testis, TSS remains the only viable option for men aiming for natural conception in the future [9].

The data regarding fertility following TSS are limited, but sperm parameters do not seem to exhibit significant changes. The most comprehensive study of TSS investigating sperm parameters in men undergoing surgery for benign lesions observed that most men were preoperatively oligospermic and asthenospermic, with no noteworthy decline postoperatively [52]. This differs from radical orchiectomy, where semen parameters invariably deteriorate, even without adjuvant therapies [49].

Previous research has indicated that GCNIS can evolve in just a few testicular lobules, leaving the remaining parenchyma with functioning spermatogenesis. If biopsies of the parenchyma surrounding the tumor indicate intact spermatogenesis and semen analysis reveals normozoospermia or oligozoospermia, around 50% of cases might result in successful paternity [16]. For such men, adjuvant radiation therapy should be deferred and replaced by routine testicular ultrasound. The latest systematic reviews seem to establish similar paternity rates in cases where TSS has been performed, with rates around 50–52% [9,28].

### 3.6. Strengths and Limitations

A limitation of our study stems from its narrative review design, which does not involve the rigorous systematic methodology inherent in systematic reviews. This format may limit the comprehensiveness and conclusiveness of our conclusions. However, we assert that our review provides valuable insights and a broad overview, serving as a platform for future systematic examinations in this field.

## 4. Conclusions

Testis-sparing surgery stands as a promising surgical approach for managing testicular tumors, providing a balance between effective oncological control and the preservation of testicular function. Particularly beneficial in cases of small, nonpalpable tumors and individuals with either bilateral tumors or solitary testicles, TSS has demonstrated low recurrence rates and nearly perfect cancer-specific survival rates.

However, these positive outcomes underline the importance of careful patient selection, taking into account individual and tumor characteristics, as well as the utility of adjuvant radiation therapy. Future large-scale and long-term studies are necessary to solidify these findings and further optimize patient selection and management strategies for TSS in testicular tumors.

## Figures and Tables

**Table 1:** Summary of patient eligibility criteria for organ-sparing surgery for testicular cancer.

Patient Factors	Tumor Factors
Normal Levels of Testosterone and Luteinizing Hormone Pre-Surgery	Tumor Size < 50% of the Testis/<2 cm
Solitary Testes	Bilateral Synchronous or Metachronous Tumors
	Indeterminate Findings on Ultrasound
	Negative Tumor Markers
	No Invasion of the Rete Testis

**Table 2:** Oncological outcomes: most relevant systematic reviews and meta-analyses over the past five years. TSS: testicular-sparing surgery. N/A: not applicable.

Author and Year	Number of Studies Included	Number of Patients	Median Follow-Up	Oncological Outcomes	Conclusions
Ory et al., 2021 [9]	32	N/A	57.8 months	N/A (No Meta-Analysis Conducted)	TSS is a safe and efficacious technique with regard to oncological control based on retrospective, non-controlled studies. TSS avoids unnecessary removal of benign testicular tissue and should be given serious consideration in cases of nonpalpable, small tumors under 2 cm.
Favilla et al., 2021 [39]	26	603	Not Specified	Local recurrence of 3.48%. Overall recurrence: 0% to 26.9% for malignancy and from 0% to 0.1% for benign lesions.	TSS was shown to be safe and practicable if used according to the specific guidelines. Urologists can consider TSS as an important means against testicular tumors in selected and well-informed patients.
Miao et al., 2021 [48]	9	320. Only Children.	Not Specified	Local recurrence 5.8% (benign rate was 70.9%)	Most of the testicular tumors in children were benign, and the most common histologic subtype was teratoma. TSS should be provided to children with benign lesions. Very low rates of tumor recurrence were observed in children with testicular tumors.
Grogg et al., 2022 [11]	32	285	38 months	Local recurrence: 13% (median 12 months), 97% disease-free after treatment for recurrence. 2% distant recurrence (median 19 months). Disease-free post-systemic treatment during a median follow-up of 52 months.	TSS should only be offered to well-informed patients with a singular testicle, excellent compliance, a singular tumor less than 2 cm located at the lower pole of the testicle, and normal preoperative endocrine function. Radical orchiectomy remains the standard of care, but future studies may support the use of TSS in selected men.
Heidenreich et al., 2023 [28]	8	252	Not Specified	Local recurrence 4–6% to 15.9%. 2–4% Distant recurrence. Cancer-specific survival close to 100%.	Oncological outcomes are excellent, with no local relapses if patients undergo adjuvant radiation therapy. The local recurrence rate might increase to 4–6% if adjuvant radiation is omitted.

## Data Availability

Not applicable.
